# Identification and Preliminary Clinical Assessment of Key Genes Related to Endoplasmic Reticulum Stress and Autophagy in Minimal Change Disease

**DOI:** 10.3390/genes17070747

**Published:** 2026-06-29

**Authors:** Ning Jiang, Guoqiang Chen, Yun Xie, Xiaofei Zhang

**Affiliations:** 1 Department of Pediatrics, The Affiliated Northwest Women’s and Children’s Hospital of Xi’an Jiaotong University Health Science Center, No. 1616, Yanxiang Road, Xi’an 710061, China; 2 Department of Medical Laboratory, The Affiliated Northwest Women’s and Children’s Hospital of Xi’an Jiaotong University Health Science Center, No. 1616, Yanxiang Road, Xi’an 710061, China

**Keywords:** endoplasmic reticulum stress, autophagy, *LIG4*, *ZRANB3*, bioinformatics

## Abstract

**Background:** Minimal change disease (MCD) is a leading cause of childhood nephrotic syndrome. Endoplasmic reticulum stress (ERS) and autophagy are implicated in its pathogenesis, but the precise mechanisms remain unclear. This study aimed to identify ERS and autophagy-related key genes (ERS-RGs and ARGs) in MCD using bioinformatic and experimental approaches. **Methods:** Transcriptomic data from GSE216841 and GSE246206 were analyzed. ERS-RGs and ARGs were obtained from prior literature. Candidate genes were selected by integrating weighted gene coexpression network analysis and differential expression analysis. Feature genes were identified via protein–protein interaction network analysis and machine learning (Least Absolute Shrinkage and Selection Operator and Boruta). Key genes were validated by expression analysis and receiver operating characteristic evaluation. A multilayer perceptron (MLP) model was constructed, and regulatory networks, immune infiltration, and chemical compound prediction were analyzed. The expression levels of the identified key genes were preliminarily assessed in peripheral blood samples using reverse transcription-quantitative polymerase chain reaction (RT-qPCR). **Results:** *LIG4* and *ZRANB3* were identified as key genes, both significantly downregulated in the MCD group, and the gene-based MLP model effectively predicted MCD probability. Overall, 13 significantly different immune cell types (e.g., CD56^+^ natural killer and activated dendritic cells) were detected. Regulatory networks (transcription factor-messenger RNA (mRNA) and long non-coding RNA-microRNA-mRNA) and 8 common chemical compounds (e.g., bisphenol A, acetaminophen) targeting these genes were predicted. Notably, peripheral blood RT-qPCR analysis revealed significant *LIG4* and *ZRANB3* downregulation, suggesting a systemic expression signature. **Conclusions:** *LIG4* and *ZRANB3* are key genes associated with ERS and autophagy in MCD, providing insights for diagnosis and targeted therapy.

## 1. Introduction

Among children, 70–90% of idiopathic nephrotic syndrome cases result from minimal change disease (MCD), a leading cause of the condition [1]. Clinically characterized by proteinuria and edema [2], MCD typically responds to glucocorticoids, although frequent relapses may require steroid-sparing immunosuppression [3]; steroid-resistant cases can progress to focal segmental glomerulosclerosis (FSGS) [4]. Histologically, MCD shows no abnormalities by light microscopy but reveals podocyte foot process effacement on electron microscopy [5]. While immune dysregulation and podocyte injury are believed to disrupt glomerular filtration barrier integrity [6], the precise molecular mechanisms driving podocyte damage in MCD remain incompletely understood.

Endoplasmic reticulum stress (ERS) occurs when misfolded or unfolded proteins accumulate in the ER lumen, triggering the unfolded protein response via three canonical sensors: PERK, ATF6, and IRE1 [7,8]. ERS has been implicated in diabetic kidney-disease-related podocyte injury [9], but its role in MCD remains largely unknown.

Importantly, ERS is closely linked to autophagy. Prolonged ERS can activate autophagy as an adaptive mechanism to clear damaged organelles and protein aggregates, thereby restoring cellular homeostasis [10]. In podocytes, basal autophagy is essential for maintaining structural and functional integrity [11], and impaired autophagy has been associated with podocyte loss in various glomerular diseases [12]. Supplementary Figure S1 summarizes the proposed ERS–autophagy interplay in MCD podocytes.

Accumulating evidence reveals complex crosstalk between ERS and autophagy [10]. Notably, the DNA damage response (DDR) pathway intersects with ERS and autophagy: unresolved ERS can induce DNA damage through reactive oxygen species accumulation, whereas DDR deficiency may exacerbate ERS-driven apoptosis [13]; autophagy also clears damaged DNA repair proteins, while DDR factors modulate autophagic flux under genotoxic stress [14,15].

This study focuses on two DDR-related candidate genes—*LIG4* and *ZRANB3*. *LIG4*, a core non-homologous end-joining factor, is involved in maintaining genomic stability under ERS conditions [13], while *ZRANB3*, an endonuclease involved in replication stress response, may influence cell fate through the p53–autophagy axis [16,17]. Although their classical functions are in DNA repair, emerging evidence suggests their involvement in an integrated stress response network that regulates podocyte homeostasis, potentially linking ERS, autophagy, and immune dysregulation in MCD.

However, the precise interplay among these pathways in MCD pathogenesis remains poorly understood [18], warranting a comprehensive investigation. Therefore, in this study, we employed a combination of bioinformatic and experimental approaches, including differential expression, weighted gene coexpression network analysis (WGCNA), protein–protein interaction (PPI) networks, and machine learning, to identify hub genes associated with ERS and autophagy in MCD. Subsequent analyses of immune infiltration, regulatory networks, and chemical compound prediction revealed potential diagnostic biomarkers and potential therapeutic targets.

## 2. Materials and Methods

### 2.1. Data Extraction

The transcriptome profiling datasets were derived from the Gene Expression Omnibus (GEO) database (https://www.ncbi.nlm.nih.gov/geo/, accessed on 24 September 2024). GSE216841(GPL20301 platform), comprising 14 MCD patient renal glomerular tissue specimens (MCD group) and 8 control renal glomerular tissue specimens (control group), served as a training set. The validation dataset was utilized from the GEO database (GSE246206, GPL20301), which included 12 MCD and 9 control kidney tissue specimens. A total of 531 autophagy-related genes (ARGs) and 256 endoplasmic reticulum stress-related genes (ERS-RGs) were retrieved from the literature [19,20], as detailed in Supplementary Tables S1 and S2, respectively.

### 2.2. Differential Expression Analysis

Initially, the boxplot function plotted all the samples in GSE216841 for quality control. Then, the DESeq2 package (v 1.34.0) [21] was utilized to detect differentially expressed genes (DEGs) in the MCD and the control specimens within GSE216841 (*p* < 0.05 & |log_2_ fold change (FC)| > 0.5). In terms of normalization, the DESeq2 package automatically estimated the size factor of each sample using the estimateSizeFactors() function and performed library size normalization based on the negative binomial distribution. During the quality control, genes with a total read count ≤ 1 across all samples were filtered out via dds[rownames(counts(dds)) > 1] to reduce the burden of multiple testing caused by low-expression genes. The ggpubr R package (v 0.6.0) was utilized to obtain a volcano plot that highlighted the top 10 down- and up-regulated genes by |log_2_FC|. The ComplexHeatmap package (v2.18.0) generated a heatmap of the DEG expression profiles.

### 2.3. WGCNA and Detection of Candidate Genes

The intersecting genes of the ARGs and ERS-RGs were acquired by the ggvenn package (v 0.1.10). Based on the profile of intersecting genes, the GSVA package (v 1.42.0) was employed to compute single-sample gene set enrichment analysis (ssGSEA) scores by non-parametrically estimating the relative enrichment of gene sets within individual samples via cumulative distribution functions. A comparison between the ssGSEA scores of the intersecting genes across groups was performed via a Wilcoxon test (*p* < 0.05). Then, the WGCNA package (v 1.71) [22] was applied to detect sample clustering outliers. To model the gene coexpression network, the parameters were set with a minimum module size equal to 50 and a merge cut height equaling 0.15, a soft-thresholding power that fulfilled the scale-free topology criterion was observed (R^2^ > 0.85) with a near-zero mean connectivity. Then, utilizing the ssGSEA scores as phenotypes, we constructed associations between phenotypes and modules, calculated the correlation coefficient (cor) matrix between module eigenvectors and phenotype traits, and plotted the correlation heatmap with the labeled Heatmap function from the WGCNA package (v 1.71) (|cor| > 0.3, *p* < 0.05). The candidate genes were defined as the intersection between the DEGs and the genes from the two key modules, which were themselves identified by having the strongest correlations with the ssGSEA scores. This intersection was subsequently visualized by employing the ggvenn package (v 0.1.10).

### 2.4. Candidate Gene PPI Network and Enrichment Analysis

Functional enrichment analysis profoundly enhanced the comprehension of complex biological systems by identifying key themes and pathways, thereby shedding light on the molecular mechanisms driving diverse diseases and physiological processes. The clusterProfiler package (v 4.7.1.3) [23] (calculate the enrichment of differentially expressed genes in specific pathways using the hypergeometric test, and then assess significance using a permutation test) was employed to conduct Kyoto Encyclopedia of Genes and Genomes (KEGG) and Gene Ontology (GO) enrichment analyses (*p* < 0.05). The GO analysis results displayed the most significant 5 pathways (ranked by ascending *p*-value) from each of the 3 primary categories: cellular components (CC), biological processes (BP), and molecular functions (MF). The results were obtained using the GOplot package (v 1.0.2). This package takes GO enrichment results as input and uses chord plots to visualize the hierarchical relationships between genes and pathways, or circle plots to illustrate the expression trends and enrichment levels of genes across multiple pathways. Similarly, the KEGG pathway analysis identified the most significantly enriched 5 pathways, with the selection being based on ascending order of *p*-value. These results were obtained using the ggplot2 package (v 3.3.6). Built on the grammar of the graphics framework, this package constructs plots by mapping data variables to aesthetic attributes (e.g., position, color, size) through geometric objects (geoms) and statistical transformations. It enables layered visualization by combining data, geometric representations, coordinate systems, and faceting schemes, facilitating the creation of publication-quality customizable figures. A PPI network was constructed via the STRING database (confidence score > 0.15) to study the interactions between the candidate genes. To identify central nodes, we then leveraged the cytoHubba plugin in Cytoscape (v 3.9.0), applying five centrality algorithms (Maximal Clique Centrality, Edge Percolated Component, Betweenness, Closeness, and Stress). We compiled a list of the top 15 genes, and finally, we used the VennDiagram package (v 1.7.1) to pinpoint the candidate feature genes common across these methods.

### 2.5. Machine-Learning Algorithms and Identification of Feature Genes

Initially, a least absolute shrinkage and selection operator (LASSO) analysis was executed to select candidate feature genes into a machine-learning composite model utilizing all available samples in GSE216841. The glmnet R package (v 4.1.8) [24] was utilized for LASSO regression analysis. LASSO regression imposed sparsity constraints on coefficients via L1 regularization and shrank the coefficients of redundant features to zero to realize embedded feature selection. Tenfold cross-validation was adopted to adaptively determine the optimal penalty parameter λ (lambda.min), which prevented the model from overfitting the training data. The final gene set was defined at the minimum lambda value, yielding a minimal error rate, and these selected predictors were designated as LASSO genes. Similarly, the Boruta algorithm was utilized to obtain Boruta genes, utilizing the Boruta package (v 8.0.0). Based on the random forest algorithm, the Boruta algorithm iteratively created shadow features and compared the relative importance between the original and shadow features. It stably screened genuine predictive factors under multiple random perturbations, and its inherent ensemble mechanism exhibited anti-overfitting properties. The overlap of genes selected by the two machine-learning algorithms was identified and defined as the final set of feature genes, which was determined utilizing the ggvenn package (v 0.1.10).

### 2.6. Identification of Key Genes

GSE216841 and GSE246206 datasets were evaluated to characterize the expression profile of the identified feature genes, comparing MCD and control samples. A Wilcoxon test assessed the statistical significance of the expression (*p* < 0.05) for each gene, and the ggplot2 package (v 3.3.6) was utilized to show the results. Genes that demonstrated highly concordant expression patterns with marked differences (*p* < 0.05) between the MCD and control groups in the 2 datasets were regarded as candidate key genes for further evaluation. To assess the discriminatory power of candidate key genes between the control and MCD specimens, we constructed receiver operating characteristic (ROC) curves for both validation sets via the pROC package (v 1.18.0). This package computes the true positive rate (sensitivity) and false positive rate (1-specificity) across all possible classification thresholds, plotting sensitivity against 1-specificity to generate the ROC curve. To be designated as a key gene, a candidate was required to exhibit an area under the curve (AUC) exceeding the threshold of 0.7 in both datasets.

### 2.7. Construction of a Multilayer Perceptron (Mlp) Network

To evaluate the collective predictive performance of the identified key genes for MCD, an MLP neural network model was constructed using the neuralnet package (v 1.44.2) in R. The model architecture comprised a single hidden layer containing 3 neurons. The input layer comprised 2 nodes, while the output layer contained 2 nodes representing the binary classification outcome (MCD vs. control).

For model training, cross-entropy (ce) was employed as the error function. The backpropagation algorithm was used for weight optimization. The logistic function was applied as the activation function in the hidden layer, with the linear.output parameter set to FALSE to ensure a nonlinear transformation. A threshold of 0.01 was set as the stopping criterion for the backpropagation algorithm; training iterations ceased when the reduction in the error function fell below this value.

Notably, instead of manually setting a fixed learning rate or number of epochs, the model leveraged the default dynamic learning rate mechanism implemented in the neuralnet package, coupled with the threshold-based early stopping strategy. No explicit regularization techniques (e.g., Dropout) were applied, as model generalizability was assessed through independent evaluation on a separate validation dataset (GSE246206) rather than through internal cross-validation.

The predictive performance of the trained MLP model was first assessed in the training set (GSE216841) and subsequently validated externally in the testing set (GSE246206). Model discrimination was evaluated using receiver operating characteristic (ROC) curves generated with the PRROC package (v 1.3.1), and the AUC was calculated. A confusion matrix was plotted using ggplot2 (v 3.3.6) to visualize the classification accuracy. An AUC > 0.7 was considered indicative of good predictive performance.

### 2.8. Gene Set Enrichment Analysis and Gene Set Variation Analysis (GSVA)

Gene set enrichment analysis (GSEA) characterized the biological pathways of the key genes in MCD. The analysis queried the key genes against the c2.cp.kegg.v7.0.symbols.gmt gene set from the Molecular Signatures Database (MSigDB) (https://www.gsea-msigdb.org/gsea/msigdb, accessed on 5 October 2024). We utilized the psych package (v 2.2.9) to calculate the Spearman correlation coefficients, followed by sorting all genes by correlation coefficients from highest to lowest. Following the implementation of the GSEA processed with the clusterProfiler package (v 4.7.1.3) exploring key gene functions (*p* < 0.05), the top 10 significant pathways were generated and visualized with the enrichplot package (v 1.18.0). Meanwhile, the GSVA package (v 1.42.0) calculated the ssGSEA scores for the KEGG pathways in MCD and control samples with the same reference gene set. The limma package (v 3.52.4) [25] compared the differences in KEGG pathway scores between different samples in the MCD and control groups (*p* < 0.05, |t| > 3.0). The cor function from the psych package (v 2.2.9) (|cor| > 0.3, *p* < 0.05) was utilized to study the correlation between the key genes and the significantly differential pathways. The obtained results were shown by the ggplot2 package (v 3.3.6).

### 2.9. Immune Infiltration Analysis

By deciphering the complex interplay between key genes and the immune landscape, immune infiltration analysis delineated various immune cell subsets. These insights helped predict therapeutic efficacy and paved the way for designing targeted immunotherapies. The ssGSEA algorithm within the GSVA package (v 1.42.0) calculated the enrichment scores for 28 kinds of immune-infiltrating cells [26] in samples in GSE216841. The ssGSEA algorithm estimates the relative abundance of each immune cell subset by quantifying the coordinated overexpression of marker genes specific to that cell type. Disparities in the proportion of immune cells between the control and the MCD groups were evaluated by the Wilcoxon test (MCD vs. control) (*p* < 0.05). The obtained results were then presented using the ggplot2 package (v 3.3.6). The interplay between key genes and immune cells was evaluated by Spearman correlation analysis across the GSE216841 dataset (|cor| > 0.3, *p* < 0.05). The correlations were subsequently depicted using a combination of the corrplot (v 1.18.0) and ggplot2 (v 3.3.6) packages.

### 2.10. Regulatory Network Analysis

Molecular regulatory networks are fundamental to deciphering the principles of gene regulation in cellular systems and to elucidating the pathogenic mechanisms underlying related diseases. Initially, ChEA3 (https://amp.pharm.mssm.edu/ChEA3, accessed on 5 October 2024) was executed to determine the transcription factors (TFs) governing the expression of the key genes. Subsequently, the microRNA Target Prediction Database (miRDB) (https://www.mirdb.org) was leveraged to forecast microRNA (miRNAs) with regulatory roles. The starBase database was employed to predict long non-coding RNAs (lncRNA) potentially associated with the candidate genes (http://starbase.sysu.edu.cn/index.php, accessed on 5 October 2024) (clipExpNum > 25). Eventually, the visualization of the regulatory network, including the relationship of TF-mRNA and lncRNA-miRNA-mRNA, was accomplished using Cytoscape software (v 3.9.0).

### 2.11. Chemical Compound Prediction

The Comparative Toxicogenomics Database (http://ctdbase.org/) was employed to search for potential compounds linked to key genes. According to the interaction scores, the top 20 chemical compounds of key genes were visualized. Cytoscape software was utilized to develop and visualize the chemical compound–mRNA interaction network (v 3.9.0).

### 2.12. Reverse Transcription-Quantitative Polymerase Chain Reaction

To preliminarily explore the expression of key genes in clinical samples, RT-qPCR experiments were conducted. Peripheral blood specimens were retrieved from 5 newly diagnosed pediatric patients with MCD who had not yet received corticosteroid therapy and were hospitalized in the Department of Pediatrics of the Affiliated Northwest Women’s and Children’s Hospital of Xi’an Jiaotong University Health Science Center. MCD diagnosis was established clinically based on the following criteria: (1) nephrotic syndrome presentation (massive proteinuria, hypoalbuminemia, edema); (2) rapid and complete remission following standard corticosteroid therapy (e.g., within 4 weeks of initiation); and (3) exclusion of secondary causes and other glomerular pathologies through comprehensive laboratory and imaging evaluations. This diagnostic approach aligns with the widely accepted clinical practice guidelines for childhood nephrotic syndrome (e.g., Kidney Disease: Improving Global Outcomes guidelines or relevant pediatric nephrology guidelines). Meanwhile, 5 age- and sex-matched healthy children who went through routine physical examinations at the health check-up center of our hospital during the same period were recruited as the control group. Children in the control group had no history of kidney disease, autoimmune disorders, or recent infections. Written consent was provided by either the legal guardians or the parents of the children. This work was authorized by the Hospital Ethics Committee (Approval No. L2023-009).

Peripheral blood mononuclear cells (PBMCs) were isolated from the collected peripheral blood samples. Total RNA was extracted from the isolated PBMCs using TRIzol reagent (Vazyme Biotech, Nanjing, China). RNA concentration was determined via NanoPhotometer N50. Subsequently, 2 μg of total RNA was reverse-transcribed into cDNA using a Hifair^®^ III; 1st Strand cDNA Synthesis SuperMix for qPCR (Yeasen, Shanghai, China) following the manufacturer’s protocol. RT-qPCR was performed on a CFX Connect Real-time Quantitative Fluorescence PCR Instrument (BIO-RAD, Hercules, CA, America) using 2× Universal Blue SYBR Green qPCR Master Mix (Servicebio, Wuhan, China). Each 10 μL reaction contained 3 μL of cDNA, 5 μL of SYBR Green master mix, and 0.5 μL each of forward and reverse primers. The thermocycling conditions were as follows: initial denaturation at 95 °C for 1 min, followed by 40 cycles of denaturation at 95 °C for 20 s, annealing at 55 °C for 20 s, and extension at 72 °C for 30 s. A melting curve analysis was performed to verify amplicon specificity. All reactions were run in triplicate. The expression levels of the key genes between the control and the MCD specimens were obtained by 2^−ΔΔCt^, and the expression differences of these genes were calculated via Student’s t-test (*p* < 0.05). Subsequently, Graphpad Prism (v 8.0) was utilized to conduct the statistical analysis and obtain visualization. The accession numbers for the human genes analyzed are as follows: *LIG4* (NM_002312.3), *ZRANB3* (NM_032143.4), and *GAPDH* (NM_001256799.3). Supplementary Table S3 lists the primer details. The differing tissue origins limit this analysis to an exploratory clinical correlation rather than direct biological validation of the glomerular transcriptomic findings.

### 2.13. Statistical Analysis

All statistical analyses were conducted using the R environment (v 4.2.2). Differential analysis between groups was executed utilizing the Wilcoxon test or Student’s *t*-test; *p* < 0.05 was considered statistically significant.

## 3. Results

### 3.1. Detection of 133 Candidate Genes

**Figure 1:**
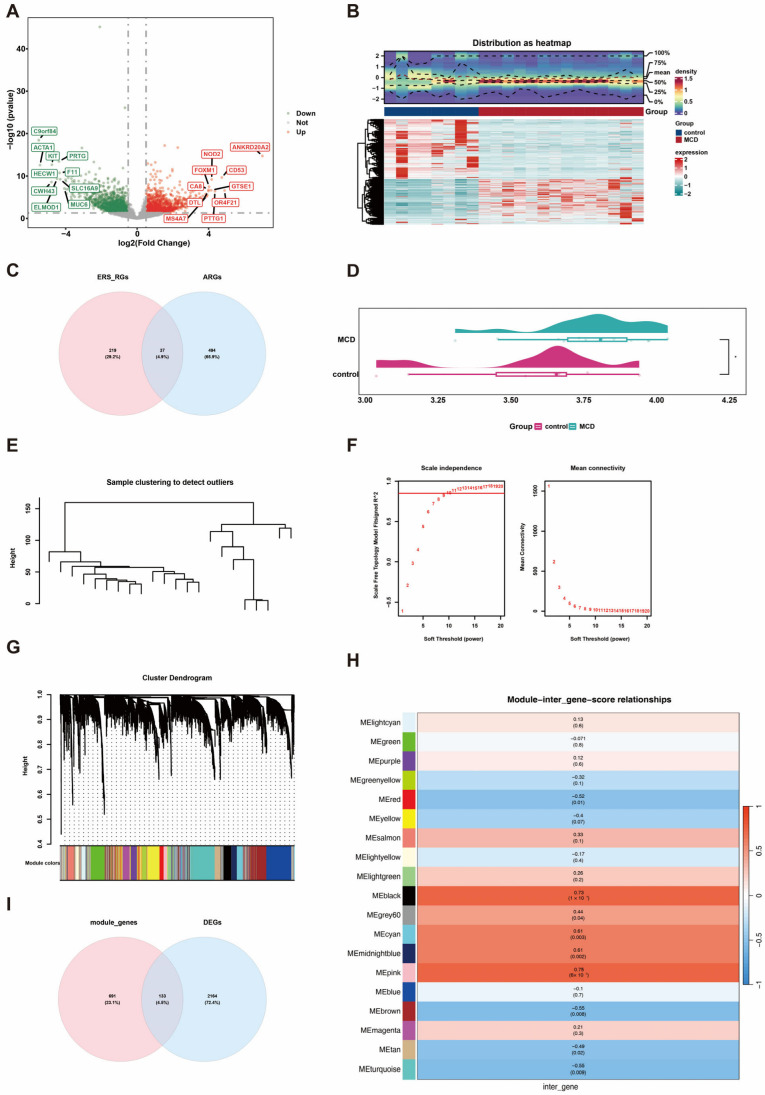
**Identification of 133 candidate genes**. (**A**) Volcano plot revealing the downregulated and up-regulated genes in minimal change disease (MCD) compared with the control group. (**B**) Heat map revealing the distribution of downregulated and up-regulated genes. (**C**) A total of 37 intersecting genes were identified from autophagy-related genes (ARGs) and endoplasmic reticulum stress-related genes (ERS-RGs). (**D**) The single-sample gene set enrichment analysis (ssGSEA) scores of intersecting genes exhibited marked differences between the control and the MCD groups (*p* < 0.05). The scattered points in the figure represent individual data points. * indicates *p* < 0.05 (**E**) Weighted gene coexpression network analysis (WGCNA) identified no outliers (sample clustering to detect outliers) with an optimal soft threshold of 10. (**F**) Scale independence and mean connectivity. The red line indicates the selected value of the scale-free fitting index. (**G**) 19 gene modules were obtained. The bottom “Module colors” color bar matches the identified co-expression gene modules; each color denotes a separate module, while the grey module includes unassigned genes that do not belong to any defined module. (**H**) 824 module genes in the key modules were selected for further evaluation. (**I**) A total of 133 candidate genes were identified through the intersection of differentially expressed genes (DEGs) and module genes.

Initially, the boxplot displayed the quality control results of the different groups in GSE216841 (Supplementary Figure S2). Differential expression analysis identified 2297 DEGs, with 1274 downregulated and 1023 up-regulated genes in the MCD specimens relative to the control specimens (Figure 1A,B). Subsequently, 37 intersecting genes were identified from ARGs and ERS-RGs (Figure 1C). Comparative analysis revealed distinct ssGSEA scores for the intersecting genes between the MCD and the control groups (*p* < 0.05) (Figure 1D), suggesting that ARGs and ERS-RGs markedly contribute to MCD development. Following this, WGCNA identified no outliers (Figure 1E) with an optimal soft threshold of 10 (Figure 1F), and then 19 gene modules were obtained (Figure 1G). The key modules with the MCD showed the strongest positive correlation (MEpink, cor = 0.75, *p* = 6 × 10^−5^) and negative correlation (MEbrown, cor = −0.55, *p* = 0.008) were obtained, and 824 module genes in the key modules were selected for additional evaluation (Figure 1H). A final set of 133 candidate genes was obtained from the overlap of the genes and the DEGs within the key modules (Figure 1I).

### 3.2. Functional Analysis of 133 Candidate Genes

**Figure 2:**
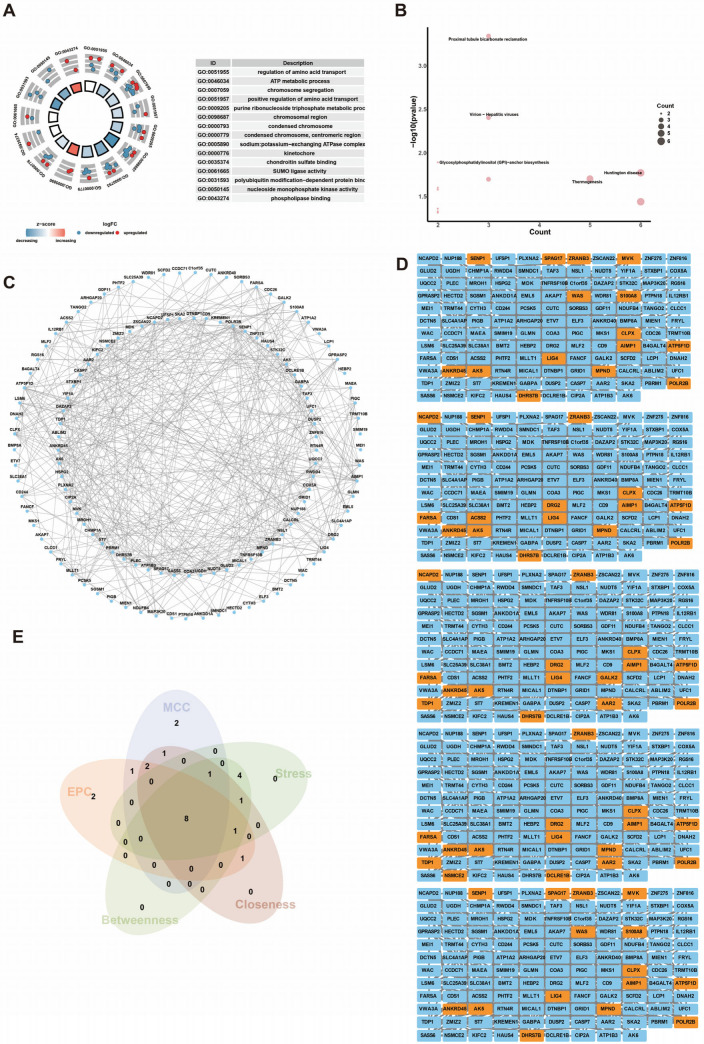
**Functional analysis of 133 candidate genes.** (**A**) The top 5 pathways in each of its 3 main categories. (**B**) 12 Kyoto Encyclopedia of Genes and Genomes (KEGG) signaling. (**C**) Protein–protein interaction (PPI) network of candidate genes. (**D**) Top 15 genes of each algorithm. (**E**) A sum of 8 candidate feature genes was obtained by taking the intersection of 5 algorithms. The blue nodes represent candidate genes, and the orange nodes are the top15 genes for each algorithm.

GO enrichment analysis indicated that the candidate genes were significantly associated with 192 signaling pathways, encompassing 147 BPs, 28 CCs, and 17 MFs. The top 5 pathways in each of its 3 main categories, comprising BPs (like regulation of amino acid transport), CCs (like chromosomal region), and MFs (like chondroitin sulfate binding), as well as 12 KEGG signaling pathways (like proximal tubule bicarbonate reclamation) (Figure 2A,B and Supplementary Tables S4 and S5). Moreover, the PPI network of candidate genes indicated numerous proteins interacting with others (Figure 2C), in which *ATP5F1D* had 20 interactions with others, such as *MLF2* and *CLPX*. After that, the top 15 genes of each algorithm were obtained (Figure 2D). The overlapping results from the five distinct algorithms yielded a final set of 8 candidate feature genes (Figure 2E).

### 3.3. Identification, Assessment of Expression, and Predictive Performance Analysis of Key Genes

**Figure 3:**
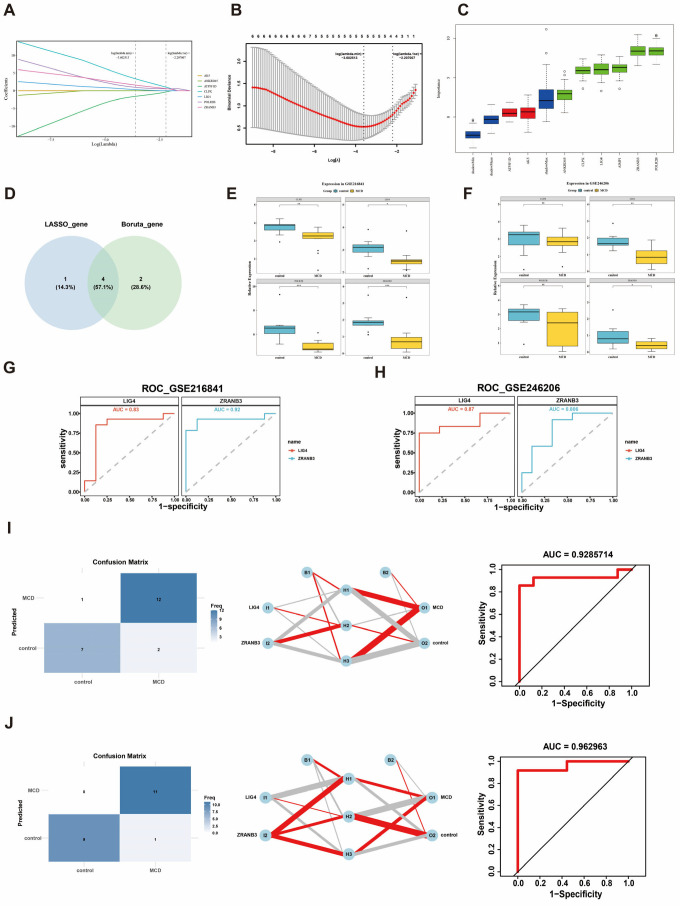
**Identification, expression verification, and predictive efficiency analysis of key genes.** (**A**) The result with the smallest error (log (lambda min) = −3.602513) was selected as the final least absolute shrinkage and selection operator (LASSO) analysis result. (**B**) The 5 LASSO genes related to MCD were obtained by the LASSO algorithm. Red dots denote the mean binomial deviation obtained from 10-fold cross-validation at each Log(λ) value. (**C**) A sum of 6 Boruta genes was picked out by the Boruta algorithm. (**D**) The intersection of genes obtained by these 2 algorithms was taken to obtain 4 feature genes, comprising *POLR2B*, *CLPX*, *LIG4*, and *ZRANB3*. Each box plot corresponds to an individual gene. Green dots indicate features confirmed important by the Boruta algorithm, blue ones represent tentative genes assigned by Boruta, and red ones denote genes classified as irrelevant by the algorithm. (**E**) Gene expression analysis in GSE216841, *LIG4*, and *ZRANB3* exhibited significant disparities observed between groups (*p* < 0.05). Blue represents the control group and yellow represents the MCD group. (**F**) Gene expression analysis in GSE246206, *LIG4*, and *ZRANB3* exhibited significant disparities observed between groups (*p* < 0.05). Blue represents the control group and yellow represents the MCD group. (**G**) The area under the curve (AUC) values of *LIG4* and *ZRANB3* were above 0.8 in GSE216841. The dashed line represents the diagonal reference line for the random classifier (AUC = 0.5). (**H**) AUC values of *LIG4* and *ZRANB3* were above 0.8 in GSE246206. The dashed line represents the diagonal reference line for the random classifier (AUC = 0.5). (**I**) The MLP model in the training set was constructed, in which the AUC equaled 0.9285714, indicating a good prediction effect. Colored lines illustrate the sign and magnitude of inter-layer neuronal connection weights: red for positive weights and gray for negative weights, with line thickness proportional to weight magnitude. The black diagonal in the ROC plot is the reference line for a random classifier (AUC = 0.5). (**J**) The MLP model was also validated with a good prediction effect for the testing set by AUC > 0.7. Colored lines illustrate the sign and magnitude of inter-layer neuronal connection weights: red for positive weights and gray for negative weights, with line thickness proportional to weight magnitude. The black diagonal in the ROC plot is the reference line for a random classifier (AUC = 0.5). ns represents *p* > 0.05, * represents *p* < 0.05, ** represents *p* < 0.01, and *** represents *p* < 0.001.

In this research, the optimal penalization coefficient for LASSO analysis (log(lambda) = −3.602513) was determined based on the minimum cross-validation error, thereby defining the final set of key genes identified through our integrated machine-learning approach. The LASSO algorithm yielded 5 genes associated with MCD (Figure 3A,B). A subsequent feature selection using the Boruta algorithm identified 6 additional genes (Figure 3C). The consensus feature genes were defined as the overlap of the outputs from the two machine-learning algorithms, resulting in 4 final candidates, comprising *POLR2B*, *CLPX*, *LIG4*, and *ZRANB3* (Figure 3D). Analysis of the gene expression data showed that *LIG4* and *ZRANB3* exhibited consistent patterns in both cohorts, with statistically discernible expression disparities between the comparative groups (*p* < 0.05) (Figure 3E,F). Additionally, in GSE216841 and GSE246206, AUC values of *LIG4* and *ZRANB3* were both above 0.8 (Figure 3G,H). Therefore, *LIG4* and *ZRANB3* were designated as key genes. Additionally, the MLP model in the training set was constructed, in which the AUC equaled 0.9285714, indicating a good prediction effect (Figure 3I). Moreover, the MLP model was validated with a good prediction effect for the testing set by AUC > 0.7 (Figure 3J). These findings suggest that *ZRANB3* carries greater weight than *LIG4* in MCD prediction.

### 3.4. GSEA and GSVA Analyses of Lig4 and Zranb3

**Figure 4:**
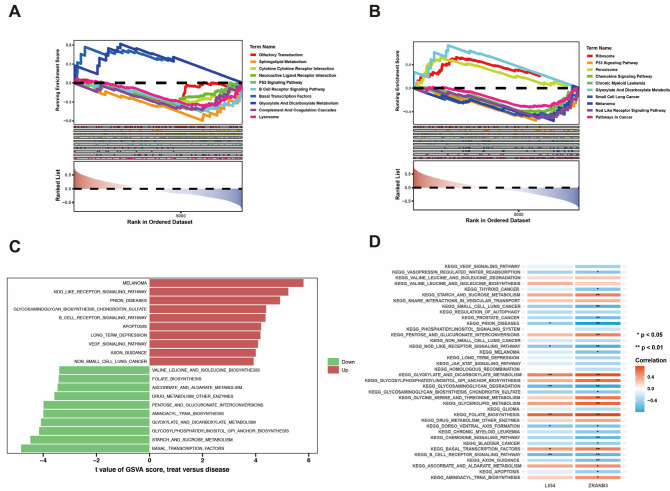
**Gene Set Variation Analysis (GSVA) and gene set enrichment analysis (GSEA) analyses of** LIG4 **and** ZRANB3 (**A**) The GSEA identified the enriched KEGG pathways for the gene *LIG4*. The bottom panel displays the sorted rank distribution of all genes; genes within the red segment are highly expressed in the transcriptome, and genes within the blue segment are lowly expressed. The dashed line denotes the zero baseline of the enrichment score (ES). (**B**) GSEA identified the enriched KEGG pathways for the gene *ZRANB3*. The bottom panel displays the sorted rank distribution of all genes; genes within the red segment are highly expressed in the transcriptome, and genes within the blue segment are lowly expressed. The dashed line denotes the zero baseline of the enrichment score (ES). (**C**) A total of 38 pathways exhibited marked differences between the MCD and normal groups in the ssGSEA scores. (**D**) Correlation between *LIG4* and *ZRANB3* genes and differential signaling pathways.

The GSEA identified 18 and 34 pathways for *LIG4* and *ZRANB3*, respectively. For the gene *LIG4*, the enriched KEGG pathways included olfactory transduction, sphingolipid metabolism, and cytokine–cytokine receptor interaction. The functional enrichment profile for *ZRANB3* ranked the ribosome, p53 signaling pathway, and chemokine signaling pathway among the top 10 most significant pathways (Figure 4A,B). Subsequently, a total of 38 pathways exhibited marked differences in the ssGSEA scores between the MCD and normal groups, including melanoma, NOD-like receptor signaling pathway, and prion diseases (Figure 4C and Supplementary Table S6). The correlation between key genes and differential signaling pathways revealed that *LIG4* and *ZRANB3* were markedly positively associated with folate biosynthesis (|cor| > 0.3, *p* < 0.01) (Figure 4D and Supplementary Table S7). The pathway with the highest negative correlation with the *LIG4* gene was glycosaminoglycan degradation (cor = −0.6, *p* < 0.01). The pathway with the highest negative correlation with the *ZRANB3* gene was prion disease (cor = −0.8, *p* < 0.01).

### 3.5. Immune Infiltration Analysis of Key Genes

**Figure 5:**
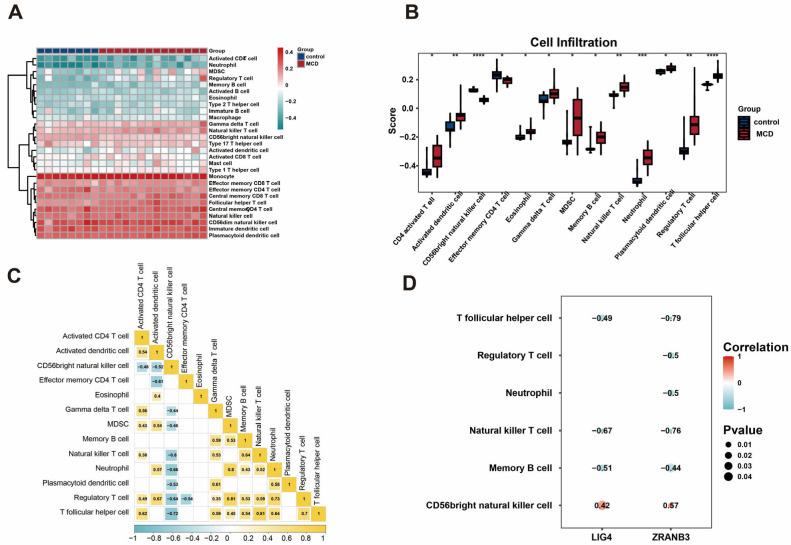
**Immune infiltration analysis of key genes.** (**A**) The estimated proportion of 28 distinct kinds of immune cells across specimens in GSE216841. (**B**) The analysis of immune-infiltrating cells revealed notable differences in the enrichment scores of 13 cell types. * represents *p* <0.05, ** represents *p* < 0.01, *** represents *p* < 0.001, and **** represents *p* < 0.0001. (**C**) Analysis of associations among immune cell differences. (**D**) Correlations between immune cells and *LIG4* or *ZRANB3*.

Analysis of the immune microenvironment, a key therapeutic target, characterized the proportions of 28 types of immune cells (Figure 5A) in the enrichment of 13 specific subsets within the GSE216841 dataset. A substantial decrease in the proportion of immune cell types, such as the CD56^bright^ natural killer and the effector memory CD4 T cells, was observed in the MCD group (*p* < 0.05). Conversely, the same group showed substantially increased proportions of activated dendritic cells, activated CD4 T cells, and neutrophils (Figure 5B). A marked positive correlation was found between the NKT and the T follicular helper cells (r = 0.81, *p* = 1.60 × 10^−5^), underscoring the remodeled immunological landscape in MCD characterized by dysregulated immune cell maturation. A pronounced negative correlation was found between the T follicular helper and the CD56^bright^ natural killer (NK) cells (*r* = −0.72, *p* = 2.41 × 10^−4^), highlighting an inverse cellular relationship between these cell types (Figure 5C and Supplementary Table S8). Interestingly, both *LIG4* (cor = 0.42, *p* = 4.99 × 10^−2^) and *ZRANB3* (cor = 0.57, *p* = 5.53 × 10^−3^) displayed the most significant positive correlation with CD56^bright^ NK cells. *LIG4* exhibited the most significant negative correlations with the NKT cells (cor = −0.67, *p* = 6.61 × 10^−4^) and *ZRANB3* exhibited the strongest negative correlations with the follicular helper T cells (cor = −0.79, *p* = 1.49 × 10^−5^) (Figure 5D and Supplementary Table S9). These insights emphasize the balance of immune cell subsets in MCD, guiding targeted immunotherapies. They contribute to early diagnosis and personalized medicine approaches based on unique immune signatures.

### 3.6. Chemical Compound Prediction and Regulatory Network Analysis

**Figure 6:**
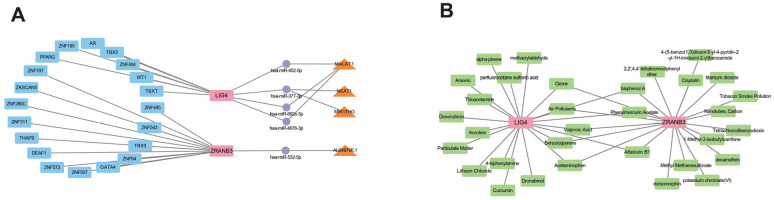
**Chemical compound prediction and regulatory network analysis.** (**A**) The regulatory network. Blue nodes represent transcription factors (TFs), pink nodes indicate hub genes, purple nodes correspond to miRNAs, and orange nodes denote lncRNAs. (**B**) The chemical compound–messenger RNA (mRNA) regulatory network indicated that a sum of 8 agents was designated as common chemical compounds for *LIG4* and *ZRANB3*. Green nodes represent predicted drugs, and pink nodes stand for key genes.

The regulatory network, including the relationships of lncRNA-miRNA-mRNA and TF-mRNA, is illustrated in Figure 6A. In detail, the TF-mRNA regulatory network indicated relationships such as *ZNF195*-*LIG4*, *TBX5*-*LIG4*, and *ZNF84*-*ZRANB3*. In addition, the lncRNA-miRNA-mRNA regulatory circuitry indicated relationships such as MALAT1-hsa-miR-452-5p-*LIG4*, MALAT1-hsa-miR-892c-3p-*LIG4*, and AL049796.1-hsa-miR-532-5p-*ZRANB3*. This comprehensive analysis provides useful insights into the molecular underpinnings of the conditions under study and may guide future therapeutic strategies. The chemical compound–mRNA regulatory network indicated that a sum of 8 chemical compounds (such as bisphenol A, acetaminophen, and aflatoxin B1) was jointly predicted by *LIG4* and *ZRANB3* (Figure 6B). This strategy highlights the potential for tailored therapies based on key genes, offering a more nuanced understanding of the chemical compound response in MCD.

### 3.7. Exploratory RT-qPCR Analysis of Key Gene Expression in Clinical Blood Samples

**Figure 7:**
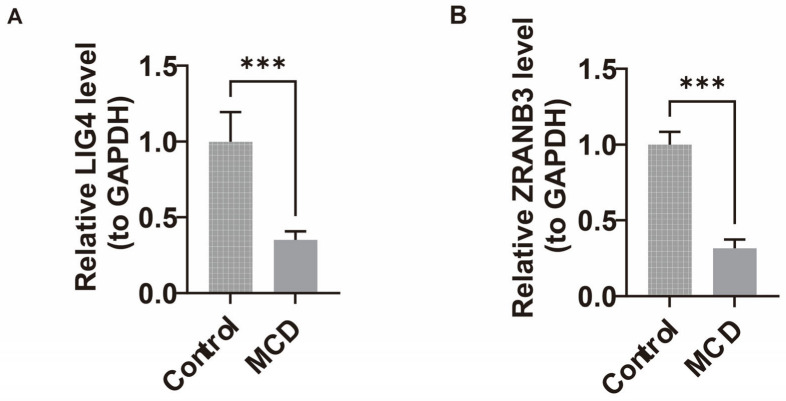
**Reverse transcription-quantitative polymerase chain reaction (RT-qPCR) experiments of key genes**. (**A**) The mRNA level of *LIG4* in MCD and control specimens (*** *p* < 0.05). (**B**) The mRNA level of *ZRANB3* in MCD and control specimens (*** *p* < 0.05).

In the RT-qPCR tests on clinical specimens, the expression of these 2 genes exhibited significant differences between the control and the MCD specimens (*p* < 0.05). In addition, *LIG4* and *ZRANB3* were downregulated in MCD samples (Figure 7A,B). This finding is consistent with the downregulation observed in glomerular tissue, suggesting a potentially detectable systemic expression change.

## 4. Discussion

The mechanisms underlying podocyte dysfunction in MCD remain poorly understood [27,28]. Herein, the integration of differential expression analysis, WGCNA, and two machine-learning algorithms (LASSO and Boruta) yielded four candidate genes, *POLR2B*, *CLPX*, *LIG4*, and *ZRANB3*. Cross-validation in independent datasets showed that *LIG4* and *ZRANB3* exhibited significant differential expression and strong diagnostic power (AUC > 0.8), with *ZRANB3* receiving the highest weight in the MLP model. RT-qPCR validation on clinical samples further confirmed their significant downregulation in MCD patients. Based on these analyses, *ZRANB3* and *LIG4* were identified as key downregulated genes in MCD. Considering their known biological functions and our pathway analysis, we hypothesize that their reduced expression may disrupt cellular homeostasis and immune balance, an effect that will require more complex experimental validation to fully establish. Notably, an MLP model constructed based on these two genes demonstrated good predictive capability for MCD, further supporting their potential as diagnostic biomarkers. Subsequent GSEA, GSVA, immune infiltration, and regulatory network analyses provided insights into the potential molecular mechanisms involving TFs (e.g., ZNF195-*LIG4*, ZNF84-*ZRANB3*) and lncRNA-miRNA-mRNA regulatory axes (e.g., MALAT1-hsa-miR-452-5p-*LIG4*).

*LIG4* encodes DNA ligase 4, a critical enzyme in non-homologous end-joining (NHEJ) repair of DNA double-strand breaks, playing a central role in maintaining genomic stability [29]. Notably, biallelic *LIG4* mutations cause a syndrome that includes renal abnormalities [29], and monoallelic loss-of-function mutations can trigger immune dysregulation via haploinsufficiency [30,31]. Beyond inherited syndromes, *LIG4* polymorphisms (e.g., single-nucleotide polymorphism rs1805388) can influence DNA repair efficiency in clinical settings [32]. In this study, *LIG4* downregulation was first identified in glomerular tissue from MCD patients through bioinformatic analysis of GEO datasets and was subsequently confirmed in peripheral blood samples from MCD patients. Based on its known biological functions, we hypothesize that reduced *LIG4* expression may impair DNA damage repair capacity and potentially interfere with its crosstalk with autophagy and ER stress [33,34]. Supporting this possibility, a kidney organoid injury model showed that the inhibition of DNA ligase 4 (encoded by *LIG4*) using SCR7 attenuated cisplatin-induced kidney injury and delayed CKD progression by modulating homologous recombination repair [35], suggesting a potential regulatory role for *LIG4* in renal injury repair. These mechanistic inferences are primarily derived from the literature knowledge and bioinformatic predictions. Our findings support *LIG4*’s role in podocyte homeostasis. However, direct functional studies must determine whether and how *LIG4* downregulation contributes to MCD-related podocyte injury.

*ZRANB3* encodes a structure-specific ATP-dependent endonuclease critical for DNA replication stress response and genome stability [36]. It resolves replication-blocking lesions by cleaving branched DNA structures, enabling repair and continuation of DNA synthesis [36,37]. *ZRANB3* operates non-redundantly with its homolog Smarcal1 in countering replication stress [38], and loss of *ZRANB3* function has been linked to replication fork instability [16]. In this study, similar to *LIG4*, *ZRANB3* downregulation was identified in glomerular tissue and confirmed in peripheral blood samples. GSEA also revealed significant enrichment of the p53 signaling pathway. p53 is known to regulate *ZRANB3*-mediated fork reversal activity, influencing cell fate under stress conditions [39]. Replication stress is an important pathological mechanism in various kidney diseases, leading to genomic instability and cellular dysfunction [40]. Based on these established associations, we hypothesize that *ZRANB3* downregulation may impair the cellular response to replication stress and potentially disrupt the crosstalk between autophagy and ER stress, thereby contributing to MCD pathogenesis. This hypothesis is derived from transcriptomic associations and literature-based inferences and requires further functional validation in podocyte-specific models.

The pathway enrichment analyses further interpreted the potential roles of *LIG4* and *ZRANB3* in MCD. *LIG4* was associated with the cytokine–cytokine receptor interaction pathway, which is closely linked to immune regulation—a long-standing focus in MCD pathogenesis, given the recognized role of T cell dysfunction and immune dysregulation [27]. As a key enzyme in NHEJ repair, *LIG4* downregulation may affect lymphocyte development and antigen receptor recombination [41], potentially disrupting immune cell homeostasis. This aligns with our immune infiltration analysis, which revealed a specific reduction in CD56^bright^ immunoregulatory NK cells and a significant positive correlation between these cells and both *LIG4* and *ZRANB3*. Conversely, *ZRANB3* was enriched in the p53 signaling pathway, central to cellular stress, DNA damage response, and apoptosis—acting as a key node connecting DNA repair, autophagy, and ERS. Additionally, GSVA showed a negative association between *LIG4* and glycosaminoglycan degradation (cor = −0.6), a pathway essential for glomerular basement membrane integrity. Collectively, these pathway signatures suggest that *LIG4* and *ZRANB3* jointly link intracellular stress responses to immune dysregulation in MCD. These findings provide a transcriptomic foundation for further studies, and direct experimental validation will be important to clarify the precise functional roles of these genes in the indicated pathways.

Our immune infiltration analysis revealed a novel association. While previous studies have documented alterations in total NK cells or the CD56^dim^ cytotoxic subset in MCD [42], we observed a specific reduction in the CD56^bright^ immunoregulatory NK cell population. Furthermore, *ZRANB3* and *LIG4* showed a significant positive correlation with this CD56^bright^ NK cell subset and negative correlations with pro-inflammatory immune subsets. These association patterns suggest that *ZRANB3* and *LIG4* link intracellular stress responses to immune dysregulation in MCD, extending previous reports of Th17/Treg imbalance in the disease [43,44]. These results reflect statistical associations between gene expression and immune cell proportions. Functional studies must determine whether these associations are causal, whether *LIG4* and *ZRANB3* directly regulate immune cell functions, and how these genes shape the immune microenvironment.

Regulatory network analysis identified upstream TFs (e.g., TBX5 for *LIG4*, ZNF84 for *ZRANB3*) and ceRNA nodes (e.g., MALAT1-miRNA-*LIG4*), providing potential targets for future therapeutic intervention. Screening for compounds that modulate ZRANB3 and *LIG4* activity represents a promising direction.

This study has limitations. First, bioinformatic analyses were performed on glomerular tissue datasets, whereas RT-qPCR validation was conducted using peripheral blood samples. This is partly because kidney tissue specimens are difficult to obtain in children with nephrotic syndrome, as most cases are highly responsive to corticosteroid therapy and do not undergo biopsies. Therefore, our validation provides only a preliminary assessment of blood-based gene expression changes rather than a direct equivalent validation of the glomerular findings. Future studies should collect paired kidney biopsies and blood samples from the same patients to directly assess the correlation of *LIG4* and *ZRANB3* expression between the two tissues. Second, the RT-qPCR validation cohort was small (*n* = 5 per group). Although the five MCD samples were obtained from treatment-naïve patients with confirmed clinical response to corticosteroid therapy, the small sample size remains a limitation that may affect findings’ robustness and generalizability. Future studies with larger cohorts, including longitudinal samples before and after treatment, are needed to validate further the clinical relevance of these genes. Third, functional experiments regarding whether *LIG4* and *ZRANB3* directly regulate ER stress, autophagy, podocyte injury, or immune dysregulation in MCD are lacking in our study. Future studies should perform loss- and gain-of-function experiments in podocyte cell lines and establish animal models to clarify the causal roles of these genes in MCD pathogenesis.

## 5. Conclusions

This bioinformatic and experimental study identifies *ZRANB3* and *LIG4* as two key downregulated genes associated with ERS and autophagy in MCD, with downregulation first identified in glomerular tissue through bioinformatic analysis and subsequently confirmed in peripheral blood samples. Their reduced expression is associated with disrupted proteostasis, altered immune infiltration (notably CD56^bright^ NK cells), and dysregulation of p53 and cytokine–cytokine receptor signaling. These findings suggest that *ZRANB3* and *LIG4* are candidate genes requiring further investigation. However, direct functional studies in podocytes and validation in glomerular tissues are required before they can be considered diagnostic biomarkers or therapeutic targets for MCD.

## Data Availability

The transcriptomic datasets analyzed during this study are provided in the GEO repository, under accession numbers GSE216841 (https://www.ncbi.nlm.nih.gov/geo/query/acc.cgi?acc=GSE216841, accessed on 24 September 2024) and GSE246206 (https://www.ncbi.nlm.nih.gov/geo/query/acc.cgi?acc=GSE246206, accessed on 24 September 2024). The clinical validation datasets developed and evaluated during the study can be provided upon reasonable request.

## Supplementary Materials

The following supporting information can be downloaded at: https://www.mdpi.com/article/10.3390/genes17070747/s1, Figure S1: Schematic diagram of endoplasmic reticulum stress and autophagy mechanisms in podocytes in minimal change disease. Figure S2: The quality control results of different groups in GSE216841; Table S1: List of 531 autophagy-related genes (ARGs); Table S2: List of 256 endoplasmic reticulum stress-related genes (ERS-RGs); Table S3: Detailed information on primers and machine testing conditions of RT-qPCR; Table S4: GO results of candidate genes; Table S5: KEGG results of candidate genes; Table S6: The scores of 38 pathways between MCD and normal groups; Table S7: The correlation between key genes and differential signaling pathways; Table S8: The correlation among immune cells; Table S9: The correlation between key genes and immune cells.
