# Supplementary figures and images for: Identification and Preliminary Clinical Assessment of Key Genes Related to Endoplasmic Reticulum Stress and Autophagy in Minimal Change Disease
Source: Genes (Basel). 2026 Jun 29;17(7):747. doi: 10.3390/genes17070747 (PMC13409386; doi:10.3390/genes17070747)

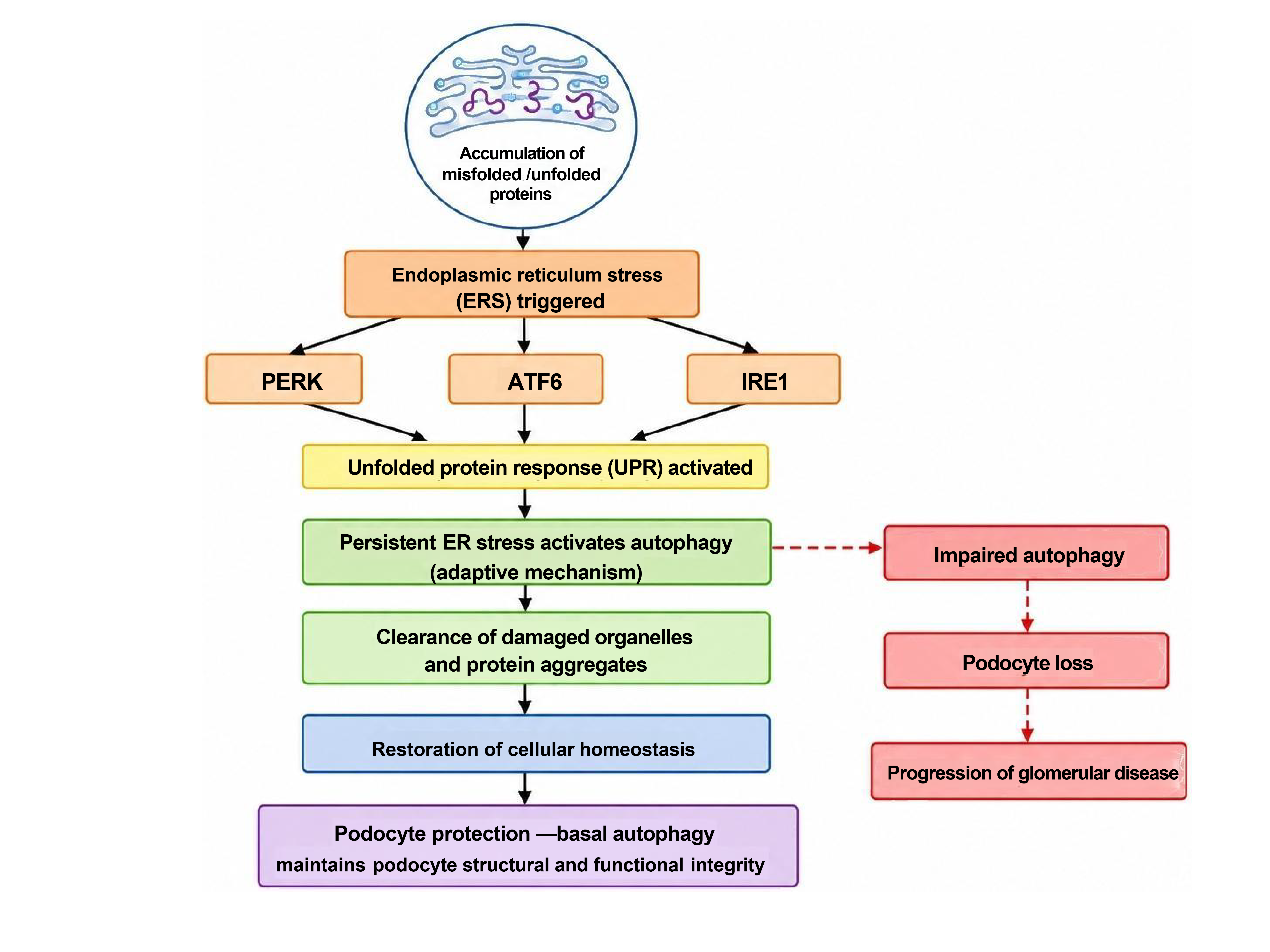

Supplement: Supplementary file 1 [file genes-17-00747-s001.zip › Supplementary Figure S1.tif]

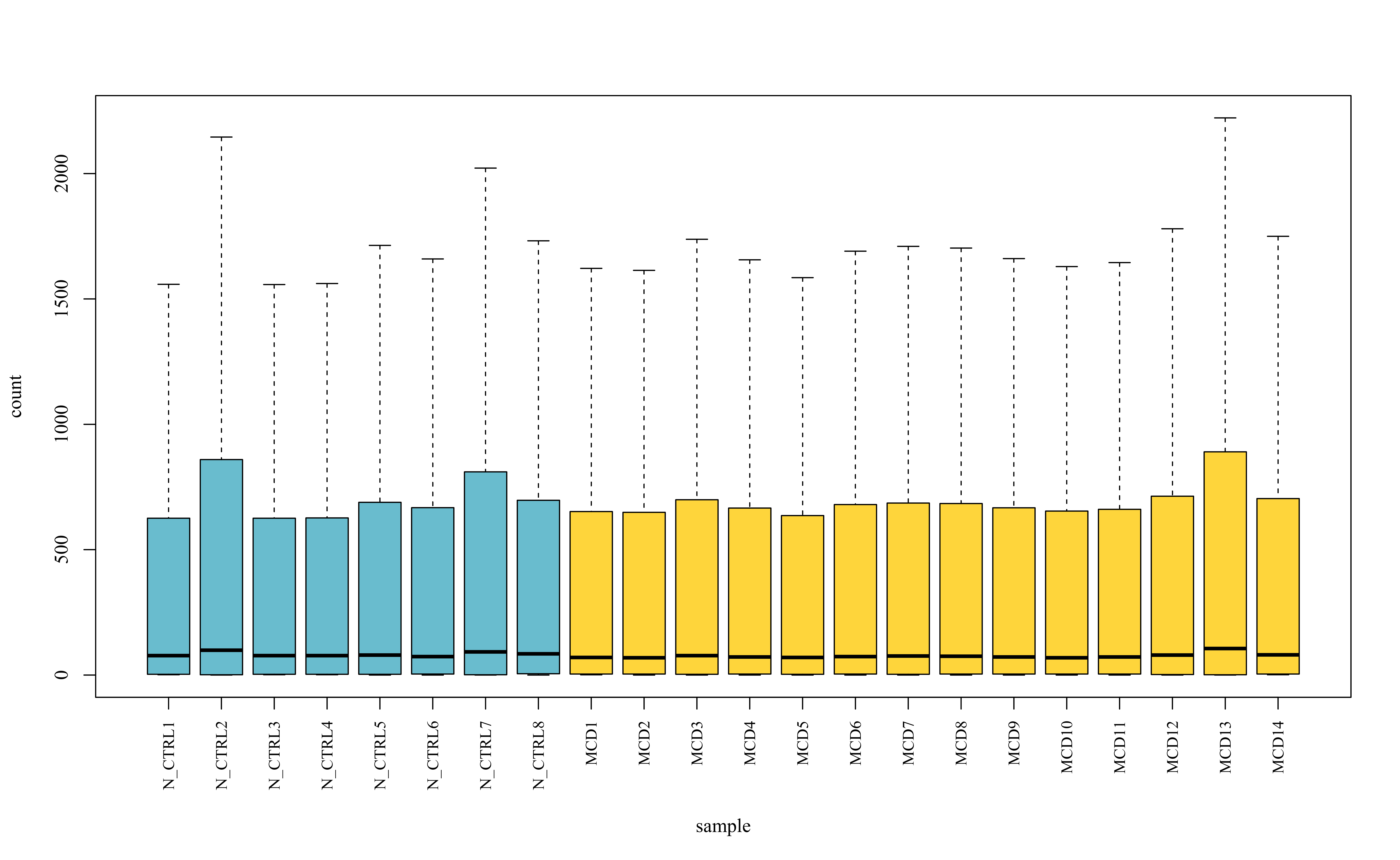

Supplement: Supplementary file 1 [file genes-17-00747-s001.zip › Supplementary Figure S2.tif]
